# Erratum: Pixel-Based Machine Learning and Image Reconstitution for Dot-ELISA Pathogen Diagnosis in Biological Samples

**DOI:** 10.3389/fmicb.2021.688832

**Published:** 2021-04-27

**Authors:** Frontiers Production Office

**Affiliations:** Frontiers Media SA, Lausanne, Switzerland

**Keywords:** dot-blot ELISA, machine learning, image analysis, serological assays, sensitivity and specificity, ROC curve, diagnostic performance

Due to a production error, two formulas were incorrectly published in the **Materials and Methods** section, subsection **Step 2: Model Selection and Supervised Training of the Classifier Algorithm**. The correct formulas are provided below.

ln [π(x)1−π(x)]=α+β1xR+β2xG+β3xB+β4xDil                              π(x)=eα+β1xR+β2xG+β3xB+β4xDil1+eα+β1xR+β2xG+β3xB+β4xDil

The publisher apologizes for this mistake. The original article has been updated.

